# Promising anticancer activity of cromolyn in colon cancer: in vitro and in vivo analysis

**DOI:** 10.1007/s00432-024-05741-2

**Published:** 2024-04-22

**Authors:** Amin Aliabadi, Mohammad Reza Haghshenas, Razie Kiani, Mohammad Reza Panjehshahin, Nasrollah Erfani

**Affiliations:** 1 Department of Pharmacology, School of Medicine, Shiraz University of Medical Sciences, Shiraz, Iran; 2 Shiraz Institute for Cancer Research, School of Medicine, Shiraz University of Medical Sciences, Shiraz, Iran; 3 Department of Immunology, School of Medicine, Shiraz University of Medical Sciences, Shiraz, Iran

**Keywords:** Cromolyn, Colon cancer, Drug repositioning, Apoptosis

## Abstract

**Purpose:**

Colon cancer is a prevalent cancer globally, representing approximately 10% of all cancer cases and accounting for 10% of all cancer-related deaths. Therefore, finding new therapeutic methods with high efficiency will be very valuable. Cromolyn (*C*), a common anti-allergic and mast cell membrane stabilizing drug, has recently shown valuable anti-cancer effects in several studies. This study was designed to investigate the anti-cancer activity of cromolyn on colon cancer in vitro and in vivo and to determine values such as selectivity index and survival effect.

**Methods:**

HT-29 (colon cancer) and MCF-10 (normal epithelial) cell lines were treated with C and Doxorubicin (DOX; Positive control). IC50 values and the effects of C and DOX on apoptosis were explored using methyl thiazole diphenyl-tetrazolium bromide (MTT) assay and Annexin V/PI Apoptosis Assay Kit. To investigate in an animal study, colon cancer was subcutaneously induced by CT26 cells (mouse colon cancer) in bulb/c mice. Mice were treated with 0.05 LD50 intraperitoneal every other day for 35 days. After the death of mice, tumor volume, tumor weight, and survival rate were evaluated.

**Results:**

C selectively and significantly suppressed the proliferation of cancer cells in a dose-dependent manner. The IC50 values for the MCF-10 and HT29 cell lines were 7.33 ± 0.78 μM and 2.33 ± 0.6 μM, respectively. Notably, the selective index (SI) highlighted that C displayed greater selectivity in inhibiting cancer cell growth compared to DOX, with SI values of 3.15 and 2.60, respectively. C exhibited higher effectiveness and selectivity in inducing apoptosis in cancer cells compared to DOX, with a significant p-value (61% vs. 52%, P-value ≤ 0.0001). Also, in mice bearing colon cancer, C reduced the tumor volume (6317 ± 1685mm^3^) and tumor weight (9.8 ± 1.6 g) compared to the negative control group (weight 12.45 ± 0.9 g; volume 7346 ± 1077) but these values were not statistically significant (P ≤ 0.05).

**Conclusion:**

Our study showed that cromolyn is a selective and strong drug in inhibiting the proliferation of colon cancer cells. Based on our results, the efficacy of C in vitro analysis (MTT assays and apoptosis), as well as animal studies is competitive with the FDA-approved drug doxorubicin. C is very promising as a low-complication and good-efficacy drug for cancer drug repositioning. This requires clinical research study designs to comprehensively evaluate its anti-cancer effects.

## Introduction

Colon cancer is one of the most common cancers worldwide, accounting for nearly 10% of all cancers and 10% of all cancer deaths (Chhikara and Parang 2023). Various factors influencing the occurrence of this disease include obesity, inactivity, age over 50, smoking, alcohol, unhealthy diet, and diabetes (Ahmed 2020). Colon cancer is preventable and can be treated when it is diagnosed early, but if it is diagnosed late, existing treatments face a challenge. Therefore, it will be very valuable to find new therapeutic methods with high efficiency (Ahmed 2020).

One promising approach in the quest for new treatments is drug repositioning, which involves repurposing existing drugs with known pharmacodynamics and pharmacokinetics for new therapeutic purposes. This strategy can significantly reduce the time and financial resources required to bring new treatments to the market (Masuda et al. 2020; Aliabadi et al. 2023).

**Fig. 1:**
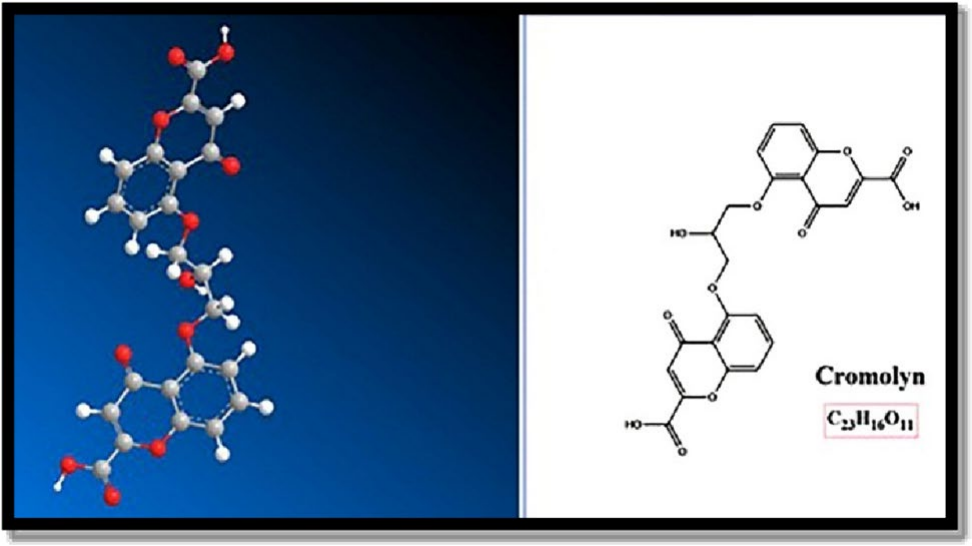
The chemical structure of Cromolyn. Cromolyn is a dicarboxylic acid derivative of bichromone. Chromone is a natural product

Cromolyn (C) is a commonly used anti-allergic drug known for its effectiveness in treating respiratory allergies. It operates by stabilizing mast cell membranes and preventing the release of histamine from immune cells (Minutello and Gupta 2020). The chemical structure of Cromolyn is shown in Fig. 1.

Recent research has revealed the potential of Cromolyn in combating cancer. Notably, mast cells have been observed infiltrating tumor tissues in breast cancer, releasing potent anticoagulants like heparin, tryptase, and chymase. Cromolyn’s ability to stabilize these mast cell membranes can lead to the formation of blood clots and hypoxia within the tumor tissue (Samoszuk and Corwin 2003).

Cromolyn’s action extends to its inhibition of glycogen synthase kinase 3 (GSK-3β), a serine-threonine kinase with diverse roles in regulating cellular pathways through protein phosphorylation. Inhibition of GSK-3β by Cromolyn prevents the degradation of p53, a key protein in controlling cell growth, and induces apoptosis as programmed cell death. This has been observed in various cancer cell types, including breast, colon, lung, liver, and oral cancers. Cromolyn increases the expression of caspase 3, a gene involved in apoptosis, decreases survivin, an anti-apoptotic protein, and promotes apoptosis in these cancer cells (Motawi et al. 2014).

Moreover, Cromolyn interferes with the function of S100P, a protein associated with tumor growth and metastasis, in pancreatic cancer cells. It inhibits the NF-κB pathway, which is crucial for the proliferation of pancreatic cancer cells, and effectively inhibits tumor growth in mouse models of pancreatic cancer (Arumugam et al. 2006).

Research in animal models of pancreatic cancer has shown that Cromolyn is more effective than gemcitabine in reducing pancreatic tumor volume. Another study, involving a 5-methyl derivative of Cromolyn, demonstrated its ability to decrease tumor weight and volume compared to a control group while significantly increasing the survival rate of the animals (Arumugam et al. 2006, 2013).

Cromolyn exemplifies drug repositioning, a strategy that involves repurposing existing drugs with known pharmacodynamics and pharmacokinetics for new therapeutic targets. This approach significantly reduces the time and cost required for researching new cancer drugs. Recent studies indicate that Cromolyn is a multi-target drug with high potency in inhibiting the proliferation of cancer cells. Additionally, Cromolyn is known for its minimal side effects, making it an attractive candidate for further investigation of its anticancer effects. Therefore, we chose Cromolyn to conduct further research into its potential anticancer properties.

Despite promising findings in various cancer types, previous studies have not explored the selectivity index (SI) of Cromolyn in inhibiting the growth of colon cancer cells or its impact on survival rates in colon cancer. This study aims to investigate these specific aspects, shedding light on Cromolyn’s potential as a therapeutic option for colon cancer.

## Materials and methods

### Materials

#### Cell lines and animals

The MCF-10 cell line, characterized as a normal epithelial cell line, and the HT-29 cell line, representing human colorectal adenocarcinoma cells, were acquired from the Pasteur Institute in Iran. Additionally, male BALB/c mice and the CT26 cell line were procured from the same source.

#### Cell culture and reagents

For cell culture, we employed DMEM medium and fetal bovine serum, both sourced from Gibco Corporation in the USA. Penicillin–streptomycin (0.1%) was acquired from Biosera Company in France. Dimethyl sulfoxide (DMSO) was prepared from materials provided by Merck in Germany, and the MTT reagent was sourced from Sigma in the USA.

#### Chemical compounds

We purchased Cromolyn from Sinadarou Pharmaceuticals in Iran and obtained Doxorubicin from Actoverco Pharmaceuticals, also based in Iran.

#### Apoptosis assay kit

To assess apoptosis, we utilized the Annexin V/PI Apoptosis Assay Kit, which was obtained from Mab-Tag GmbH Company in Germany.

#### Cell culture

In the cell culture procedure, HT-29 cells were grown in high-glucose DMEM, while MCF-10 cells were cultured in DMEM. Both culture media were supplemented with 10% fetal bovine serum (FBS) and 1% penicillin–streptomycin. The cells were maintained in a controlled environment at 37 °C with 5% CO2 for optimal growth conditions. To assess cell viability, Trypan blue staining was employed (Freshney 2015).

#### MTT assay

Cell proliferation was assessed using the MTT assay, a well-established technique. HT-29 and MCF-10 cells were seeded at a density of 10^4 cells per well in 96-well microplates, reaching 80% confluence. They were then incubated at 37 °C in a 5% CO2 environment. We added different concentrations of Cromolyn, ranging from 0.5 to 16 µM, and Doxorubicin, used as a positive control drug with concentrations of 1, 2, 4, 8, 16, and 32 nM, to the wells containing fresh media. This replacement was carried out after 24 h, while the negative control wells were filled with fresh media containing only DMSO. Following this, the microplates were incubated for 72 h in a controlled environment at 37 °C with 5% CO2. Subsequently, 10 µL of MTT reagent at a concentration of 0.5 mg/mL was added to each well, and the incubation continued in darkness for 4 h. After removing the supernatant, 150 µL of DMSO was introduced into each well to serve as a formazan solvent. The microplates were then agitated for 20 min on a shaker. Optical density (OD) at a wavelength of 570 nm was measured for each well using an ELISA reader. These experimental procedures were carried out in triplicate. The percentage of viable cells was calculated using the formula: 100—(OD treatment / OD control) × 100 (Freshney 2015).

IC50 values, representing the concentration at which cell proliferation is inhibited by 50%, were determined using CurvExpert 1.4 software. The selectivity index (SI), which assesses compound safety, was calculated using the formula: Selectivity Index = IC50 in normal cells / IC50 in cancer cells. A compound with an SI greater than 2 is considered selective (Demirgan et al. 2016; Zbakh et al. 2020).

#### Apoptosis assay by flow cytometry

To evaluate the impact of Cromolyn and DOX on apoptosis in HT-29 and MCF-10 cell lines, we utilized the Annexin V/PI Apoptosis Assay Kit. Initially, the cells were seeded in 24-well plates and allowed to incubate overnight at 37 °C in a 5% CO2 environment. The following day, the culture medium was replaced with a fresh medium containing IC50 concentrations of Cromolyn and DOX, and this incubation was sustained for 48 h. Subsequently, the cells were transferred to flow cytometry tubes and washed with PBS. In each tube, a mixture of 100 µL of binding buffer, 5 µL of Annexin-V, and 5 µL of PI was added, followed by a 20-min incubation in darkness. Following incubation, 400 µL of binding buffer was introduced, and the tubes underwent centrifugation at 400 g for five minutes. Finally, the cells were resuspended in an additional 500 µL of binding buffer before being analyzed using flow cytometry software. (Adan et al. 2017).

#### Animal study of colon cancer

For this study, we utilized a total of thirty male BALB/c mice, each within the same age range and weighing between 20 to 25 g. The CT26 cells were cultured, and subsequently, we introduced 2 × 10^6^ cells suspended in 10µL of PBS via subcutaneous injection into the left flank of three mice. Three weeks later, when the tumors had grown to a size of 1,500 mm^3^, they were surgically excised, and 5 mm^3^ sections were prepared by punching. These sections were then transplanted subcutaneously into the left side of 18 other mice.

Two weeks following the transplantation, when the tumors had reached a size of 100 mm^3^, we divided the mice into three groups, each consisting of six individuals. The Cromolyn group received Cromolyn intraperitoneal (IP) at a dose of 50 mg/kg every other day for a period of 35 days, equivalent to 0.05 LD50 of the drug. Similarly, the DOX group received DOX at a dose of 558 µg/kg IP every other day for 35 days, also equivalent to 0.05 LD50. The LD50 dose of the drug was determined based on data extracted from past articles mentioned on the https://pubchem.ncbi.nlm.nih.gov website.

The Cromolyn group received Cromolyn intraperitoneal (IP) at a dose of 50 mg/kg, equivalent to 0.05 × LD50 of the drug. Katzung and Trevor’s textbook Pharmacology states that therapeutic doses in humans are equivalent to 0.01–0.10 (mean 0.05) LD-50 in animals. Thus, we chose this dose.

The negative control group was administered normal physiological saline. Upon the death of each mouse, we removed the tumors, and a pathologist verified the tumor tissues after preparing slides (Xu et al. 2017; Aliabadi et al. 2023).

We measured the weight of the tumors using a digital scale and assessed their dimensions with calipers to determine their small and large diameters:

$${\text{Tumor volume }} = \, \left( {{\text{small diameter}}^{{2}} \times {\text{large diameter}}} \right)/{2}$$

### Statistical analysis

We determined the IC50 values using Curve Expert software version 1.4 (USA) and expressed them as the mean ± standard deviation (SD) based on a minimum of three separate experiments. Statistical analyses, including One-way ANOVA and Tukey multiple comparisons, were performed with Graph Pad Prism software version 8 (Inc; San Diego CA, USA, 2003). This software was also utilized to create the statistical graphs. A p-value below 0.05 was considered statistically significant.

## Results

### MTT assay

**Fig. 2:**
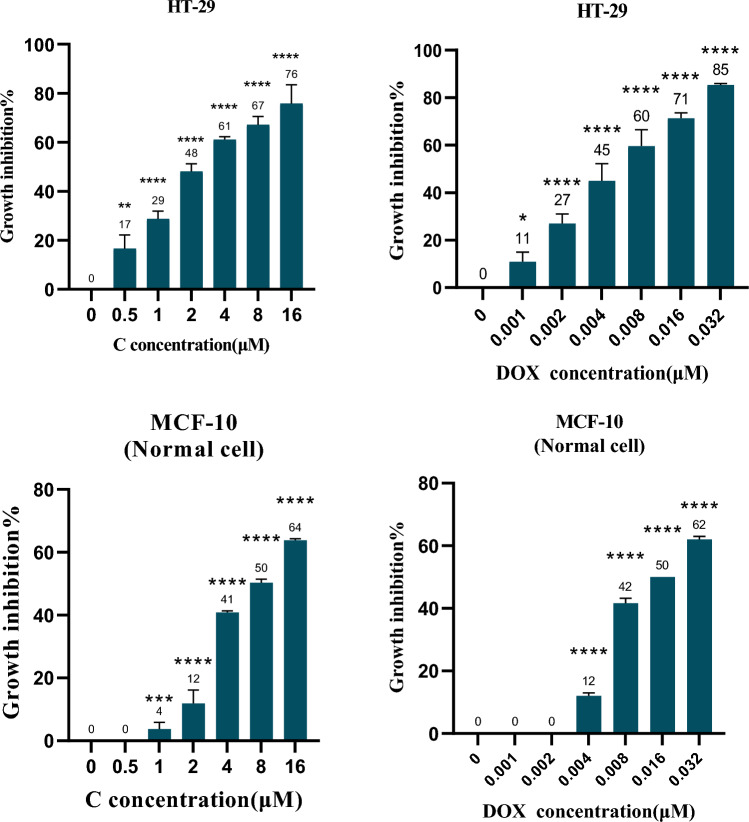
MTT assay results for HT-29 (colon cancer) and MCF-10 (normal epithelial) cell proliferation after 72-h incubation with Cromolyn (C) and Doxorubicin (DOX). The graph presents the mean ± standard deviation from three independent experiments. One-way ANOVA tests indicate a significant difference between C-treated and 5% DMSO-treated cells. C exhibited higher selectivity than DOX in inhibiting cancer cell proliferation compared to normal cells (Selectivity Index: 3.15 vs. 2.60). (*) denotes statistical significance (P-value* < 0.05), and (***) represents even higher statistical significance (P-value < 0.0001)

Cromolyn (C) exhibited selective and significant suppression of cancer cell proliferation in a dose-dependent manner after a 72-h incubation period. The IC50 values for the MCF-10 and HT-29 cell lines were 7.33 ± 0.78 μM and 2.33 ± 0.6 μM, respectively. Notably, the selective index (SI) indicated that C demonstrated greater selectivity in inhibiting cancer cell growth compared to DOX, with SI values of 3.15 and 2.60, respectively (Fig. 2).

Furthermore, in the MTT assay, it was apparent that DOX achieved a maximum inhibitory effect of 85%, while C reached a significant 76%, which is considered relatively optimal. These findings are graphically depicted in Fig. 2 and elaborated upon in Tables 1 and 2.

**Table 1:** The mean ± SD IC50 Values of Cromolyn (C) and Doxorubicin (DOX) in colon cancer and normal Cell Lines

IC50 (µM)		
	C	DOX
HT-29	2.33 ± 0.6	0.005 ± 0.001
MCF10 (Normal cell)	7.33 ± 0.78	0.013 ± 0

**Table 2:** Selectivity Index (SI) rate of Cromolyn (C) and Doxorubicin (DOX) in HT-29 cell line

Selectivity index (SI)		
	C	DOX
HT-29	3.15	2.60

### Apoptosis assay by flow cytometry

**Fig. 3:**
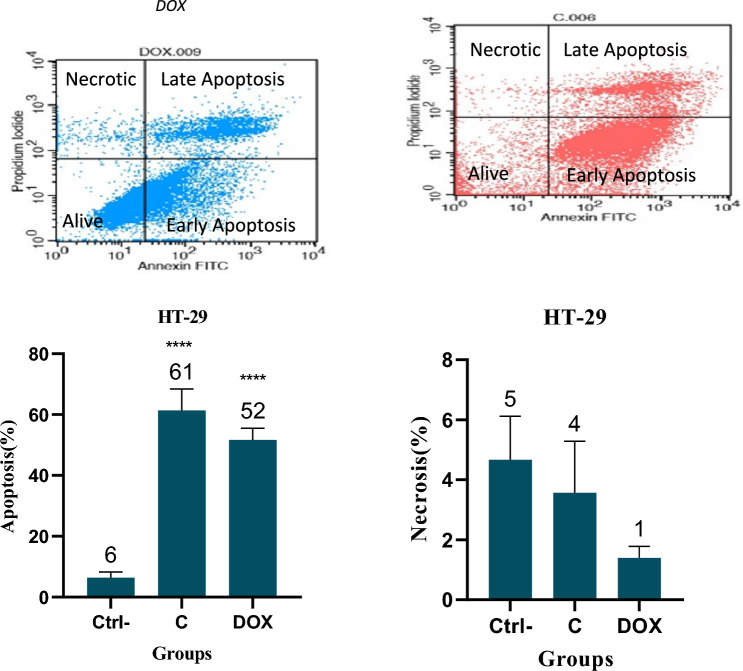
Apoptosis analysis was conducted using Annexin V/PI staining in HT-29 cells treated with Cromolyn (C) and Doxorubicin (DOX) at IC50 concentration for 48 h. Cromolyn (C) induced (61 ± 7) % apoptosis in the HT29 cancer cell line with a significant p-value (P-value ≤ 0.0001) and demonstrated higher efficacy compared to DOX in inducing apoptosis in cancer cells. The graph displays the Means ± Standard Error of the Mean (SEM) from three independent experiments. One-way ANOVA tests revealed a noteworthy difference between the control (Ctrl), Cromolyn (C)-treated, and DOX-treated cells. (****) denotes a high level of statistical significance (P-value < 0.0001)

**Fig. 4:**
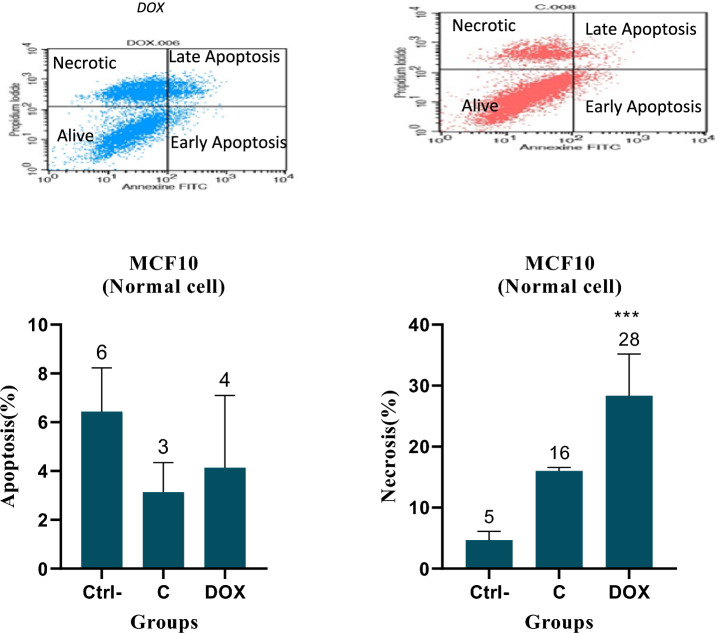
Apoptosis analysis using Annexin V/PI staining was conducted in MCF-10 (Normal) cells treated with Cromolyn (C) and Doxorubicin (DOX) at IC50 concentration for 48 h. Cromolyn (C) did not induce significant apoptosis or necrosis compared to the control group in MCF-10 (normal cells) and displayed greater selectivity than DOX. The graph illustrates the Means ± Standard Error of the Mean (SEM) based on three independent experiments. One-way ANOVA tests revealed a significant difference between the control (Ctrl), Cromolyn (C)-treated, and DOX-treated cells. (*) signifies statistical significance (P-value < 0.05)

HT-29 and MCF-10 cell lines were exposed to Cromolyn (C) and DOX at IC50 concentrations obtained from the MTT test for 48 h. Cromolyn induced apoptosis in the HT29 cancer cell line at a rate of (61 ± 7) %. Moreover, Cromolyn exhibited higher effectiveness and selectivity in inducing apoptosis in cancer cells compared to DOX, with a significant p-value (61% vs. 52%, P-value ≤ 0.0001). Notably, Cromolyn did not demonstrate significant apoptosis and necrosis in normal cells when compared to the control group (refer to Figs. 3 and 4).

### Animal study of colon cancer

**Fig. 5:**
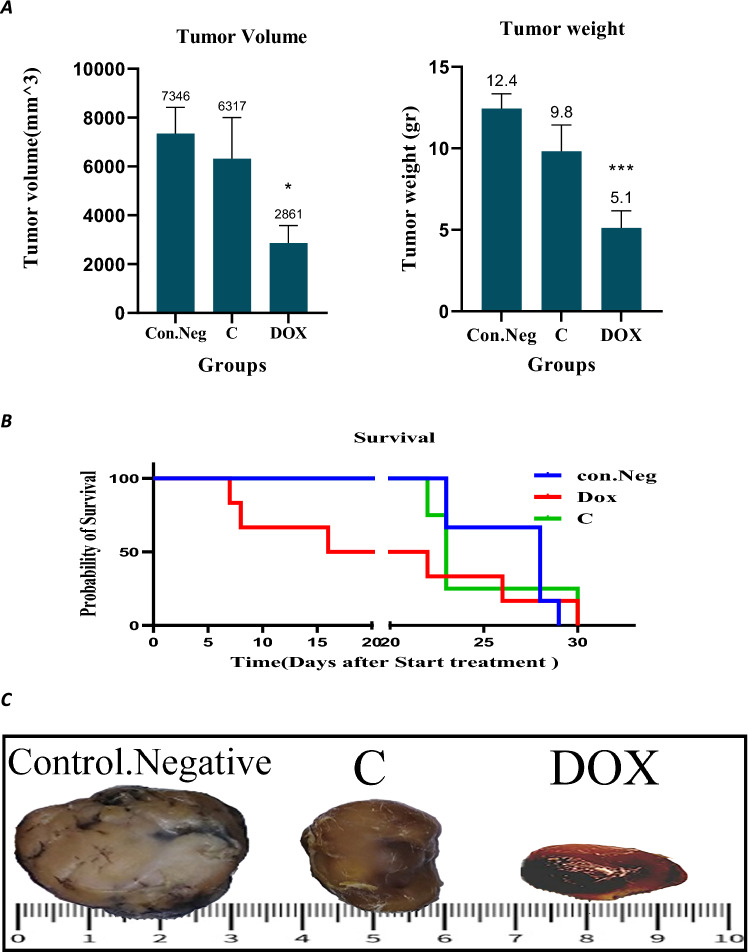
**A** Tumor Weight and Volume Comparison: The graph illustrates the mean ± SEM of tumor weight and volume in different treatment groups. Cromolyn (C) treatment reduced the tumor volume to (6317 ± 1685 mm3) and the tumor weight to (9.8 ± 1.6 g) compared to the negative control group (weight: 12.45 ± 0.9 g; volume: 7346 ± 1077 mm3). However, these differences were not statistically significant (P ≤ 0.05). **B** Survival Rates of CT26-Tumor-Bearing Mice: Survival rates of CT26-tumor-bearing mice (colon cancer) treated with Cromolyn (C) and Doxorubicin (DOX) based on the Kaplan–Meier curve. The survival analysis by the Kaplan–Meier chart did not reveal a significant difference among the three groups. **C** Tumors from Different Treatment Groups: This section displays tumors removed from various treatment groups, with one representative tumor from each treatment group shown

Next, we evaluated the anti-cancer activity of Cromolyn (C) compared to Doxorubicin (DOX) and the negative control group against subcutaneous CT-26 tumors in male BALB/c mice (refer to Fig. 5).

Treatment with Cromolyn resulted in a reduction of tumor volume to (6317 ± 1685) mm3 and tumor weight to (9.8 ± 1.6) grams when compared to the negative control group (weight: 12.45 ± 2 g; volume: 7346 ± 1077 mm3). However, these differences did not reach statistical significance (refer to Fig. 5A and C). Kaplan–Meier survival analysis did not reveal a significant distinction among the three groups (refer to Fig. 5B).

## Discussion

Cromolyn sodium (C) is widely used in the treatment of respiratory sensitivity to allergens. C stabilizes mast cell membranes and prevents the release of histamine from immune cell vacuoles (Minutello and Gupta 2020).

Our study demonstrated that cromolyn selectively, dose-dependently, and potently inhibited the proliferation of colon cancer cells (HT-29) after 72 h of incubation (IC50 less than 5). Similar inhibitory effects of cromolyn on the proliferation of various cancer cell types, including liver, larynx, and cervix, have been observed, with IC50 values around 4 μM (Motawi et al. 2014). The anticancer potential of cromolyn (IC50) has been shown to be comparable to standard drugs. However, due to its low oral absorption, there is a need to develop injectable and oral formulations with high drug absorption. In this regard, formulations of the 5-methyl derivative of cromolyn and liposomal formulations for pancreatic cancer (Kim et al. 2012; Motawi et al. 2017), as well as chitosan formulations for colon cancer, have been developed and investigated to enhance the efficiency of cromolyn, with some success (Motawi et al. 2017).

Regarding effectiveness (maximum response), the maximum effectiveness of C in inhibiting cancer cell proliferation in the MTT test is nearly equivalent to that of DOX.

To date, the Selectivity Index (SI) of cromolyn in inhibiting cancer cell growth has not been explored in previous studies. Examination of the Selectivity Index (SI) of cromolyn reveals that C is more than three times more selective in inhibiting cancer cell proliferation than normal cells and even more selective than DOX.

Cromolyn, at the IC50 concentration, induced higher apoptosis than DOX in the colon cancer cell line, and this difference was statistically significant. Fortunately, cromolyn did not cause significant apoptosis and necrosis in normal cells compared to the negative control (MCF-10).

The effect of cromolyn in inducing apoptosis at a concentration of 10 μM and an incubation period of 96 h has been observed in various types of cancer cells, including liver, larynx, and cervix, at a rate of 50–60%. By comparing the apoptosis results of the aforementioned study with our study, it appears that the maximum amount of apoptosis occurs at a concentration of 2.3 and an incubation time of 72 h, with no significant increase beyond this concentration and incubation time (Motawi et al. 2014).

Furthermore, to further investigate the effects of cromolyn, we evaluated it in an animal model of colon cancer. Cromolyn was able to reduce the tumor weight by 20% and tumor volume by 14% compared to the negative control group. However, these reductions were not statistically significant. Although the results of weight and volume reduction were not significant in our animal study, based on the promising results of the in vitro part, we hope that in our future animal studies with a new design, including increasing the number of mice, using different doses of the drug, prescribing a daily dose instead of every other day, and preparing new formulations, we can enhance the effectiveness of Cromolyn in reducing tumor weight and volume (P-value ≤ 0.05).

The survival analysis using the Kaplan–Meier test did not reveal a significant difference among the three groups. Although this drug could not improve the survival rate of mice, its effect on survival was better than that of doxorubicin. Previous experiments have shown that cromolyn can suppress tumor growth signals such as BCL-2 and s100P in the animal model of colon cancer induced by dimethylhydrazine. Additionally, its nano chitosan formulation was highly effective in reducing the number of precancerous lesions and enhancing its effectiveness in colon cancer (Motawi et al. 2014, 2017).

Research on the effects of cromolyn and its PEGylated liposome formulation in an animal model of pancreatic cancer has demonstrated that this drug can significantly reduce tumor volume (Kim et al. 2012).

Previous observations in animal models of pancreatic cancer have indicated that cromolyn outperforms gemcitabine in inhibiting pancreatic tumor volume. Additionally, another study on a 5-methyl cromolyn derivative showed that this compound was able to reduce the volume and weight of the tumor by one-third compared to the negative control group and significantly increase the survival rate of the animals (Arumugam et al. 2006, 2013). Comparing our observations with past experiments indicates that cromolyn is effective in controlling tumor growth both in vivo and in vitro, and this efficiency can be further enhanced with new formulations.

In summary, our study underscores Cromolyn as a potent and highly selective agent for combating colon cancer. Its efficacy across various assays, including MTT tests and apoptosis induction, as well as its performance in animal models, is comparable to the well-established FDA-approved drug, doxorubicin. Importantly, Cromolyn maintains a higher degree of selectivity, crucial for minimizing collateral damage to healthy cells. Cromolyn emerges as a promising candidate for low-complication cancer therapy, potentially reshaping the landscape of cancer treatment. Further research to unlock its full anticancer potential holds significant promise for future therapeutic development.

## Data Availability

The datasets generated and analyzed during the current study are available from the corresponding author upon reasonable request.

## References

[CR1] Adan A, Alizada G, Kiraz Y, Baran Y, Nalbant A (2017). Flow cytometry: basic principles and applications. Crit Rev Biotechnol.

[CR2] Ahmed M (2020). Colon cancer: a clinician’s perspective in 2019. Gastroenterol Res.

[CR3] Aliabadi A, Haghshenas MR, Kiani R, Koohi-Hosseinabadi O, Purkhosrow A, Pirsalami F, Panjehshahin MR, Erfani N (2023). In vitro and in vivo anticancer activity of mebendazole in colon cancer: a promising drug repositioning. Naunyn-Schmiedeberg’s Arch Pharmacol.

[CR4] Arumugam T, Ramachandran V, Logsdon CD (2006). Effect of cromolyn on S100P interactions with RAGE and pancreatic cancer growth and invasion in mouse models. J Natl Cancer Inst.

[CR5] Arumugam T, Ramachandran V, Sun D, Peng Z, Pal A, Maxwell DS, Bornmann WG, Logsdon CD (2013). Designing and developing S100P inhibitor 5-methyl cromolyn for pancreatic cancer therapy. Mol Cancer Ther.

[CR6] Chhikara BS, Parang K (2023). Global Cancer Statistics 2022: the trends projection analysis. Chem Biol Lett.

[CR7] Demirgan R, Karagöz A, Pekmez M, Önay-Uçar E, Artun FT, Gürer Ç, Mat A (2016). In vitro anticancer activity and cytotoxicity of some papaver alkaloids on cancer and normal cell lines. Afr J Tradit Complement Altern Med.

[CR8] Freshney RI (2015). Culture of animal cells: a manual of basic technique and specialized applications.

[CR9] Kim C-E, Lim S-K, Kim J-S (2012). In vivo antitumor effect of cromolyn in PEGylated liposomes for pancreatic cancer. J Control Release.

[CR10] Masuda T, Tsuruda Y, Matsumoto Y, Uchida H, Nakayama KI, Mimori K (2020). Drug repositioning in cancer: The current situation in Japan. Cancer Sci.

[CR11] Minutello K, Gupta V (2020) Cromolyn sodium 32491405

[CR12] Motawi TM, Bustanji Y, El-Maraghy S, Taha MO, Al-Ghussein MA (2014). Evaluation of naproxen and cromolyn activities against cancer cells viability, proliferation, apoptosis, p53 and gene expression of survivin and caspase-3. J Enzyme Inhib Med Chem.

[CR13] Motawi TK, El-Maraghy SA, ElMeshad AN, Nady OM, Hammam OA (2017). Cromolyn chitosan nanoparticles as a novel protective approach for colorectal cancer. Chem Biol Interact.

[CR14] Samoszuk M, Corwin MA (2003). Mast cell inhibitor cromolyn increases blood clotting and hypoxia in murine breast cancer. Int J Cancer.

[CR15] Xu JX, Xiong W, Zeng Z, Tang Y, Wang YL, Xiao M, Li M, Li QS, Song GL, Kuang J (2017). Effect of ART1 on the proliferation and migration of mouse colon carcinoma CT26 cells in vivo. Mol Med Rep.

[CR16] Zbakh H, Zubía E, Reyes CDI, Calderón-Montaño JM, López-Lázaro M, Motilva V (2020). Meroterpenoids from the brown alga Cystoseira usneoides as potential anti-inflammatory and lung anticancer agents. Marine Drugs.

